# TRPV6 Calcium Channel Targeting by Antibodies Raised against Extracellular Epitopes Induces Prostate Cancer Cell Apoptosis

**DOI:** 10.3390/cancers15061825

**Published:** 2023-03-17

**Authors:** Aurélien Haustrate, George Shapovalov, Corentin Spriet, Clément Cordier, Artem Kondratskyi, Lucile Noyer, François Foulquier, Natalia Prevarskaya, V’yacheslav Lehen’kyi

**Affiliations:** 1 Laboratory of Cell Physiology, INSERM U1003, Laboratory of Excellence Ion Channels Science and Therapeutics, Department of Biology, Faculty of Science and Technologies, University of Lille, 59650 Villeneuve d’Ascq, Francenatacha.prevarskaya@univ-lille.fr (N.P.); 2 FONDATION ARC, 9 rue Guy Môquet, 94830 Villejuif, France; 3 Unité de Glycobiologie Structurale et Fonctionnelle (UGSF), CNRS, UMR 8576, Université de Lille, 59000 Lille, France

**Keywords:** therapeutic antibody, TRPV6 channel, tumor targeting, apoptosis

## Abstract

**Simple Summary:**

The TRPV6 channel is upregulated in many cancers and is associated with bad prognosis. Despite this fact, no reliable tool to target TRPV6 is known so far. Two prospective antibodies have been generated capable of binding to TRPV6 and thereby transiently activating calcium currents. This transient activation of TRPV6 and increase in intracellular calcium led to the apoptosis induction of prostate cancer cells. Only cells showing no TRPV6 membrane expression avoided cell death. Thus, the activation of the TRPV6 channel *per se* is extremely efficient and is likely to be more prospective than its inhibition.

**Abstract:**

The TRPV6 calcium channel is known to be up-regulated in various tumors. The efforts to target the TRPV6 channel in vivo are still ongoing to propose an effective therapy against cancer. Here, we report the generation of two antibodies raised against extracellular epitopes corresponding to the extracellular loop between S1 and S2 (rb79) and the pore region (rb82). These antibodies generated a complex biphasic response with the transient activation of the TRPV6 channel. Store-operated calcium entry was consequently potentiated in the prostate cancer cell line LNCaP upon the treatment. Both rb79 and rb82 antibodies significantly decreased cell survival rate in a dose-dependent manner as compared to the control antibodies of the same isotype. This decrease was due to the enhanced cell death via apoptosis revealed using a sub-G1 peak in a cell cycle assay, TUNEL assay, and a Hoechst staining, having no effects in the PC3M*^trpv6−/−^* cell line. Moreover, all TUNEL-positive cells had TRPV6 membrane staining as compared to the control antibody treatment where TRPV6-positive cells were all TUNEL negative. These data clearly demonstrate that TRPV6 channel targeting using rb79 and rb82 antibodies is fatal and may be successfully used in the anticancer therapies.

## 1. Introduction

According to the French National Institute of Cancer (INCa), 384,442 new cancer cases were reported in France in 2015 with the death rate reaching 149,456 of the same year (http://lesdonnees.e-cancer.fr/, accessed on 19 May 2018). Literally, 411 persons die every day from cancer. Of them, the most mortality is from lung, colon-rectum, breast, and prostate (25.3%, 15.0%, 10.1, and 7.5% of the total number, respectively) cancers (http://lesdonnees.e-cancer.fr/, accessed on 19 May 2018). Moreover, the treatment is very expensive and has important consequences on patient health [1]. In Germany, for instance, the cost of the treatment against prostate cancer equals EUR 270,000,000 per year. Thus, cancer is a major health problem in Europe.

The TRPV6 channel is a membrane protein participating in calcium transport in the body and belongs to the superfamily of transient receptor potential (TRP) channels, subfamily vanilloid, member 6 [2]. Among all TRP channels TRPV6 is highly Ca^2+^ selective, with PCa/PNa values exceeding 100; such high Ca^2+^ selectivity is unique within the TRP superfamily (plus closely related TRPV5) and makes this channel quite distinguishable, especially in Ca^2+^-related intracellular pathways [3]. Indeed, due to its high calcium selectivity over other TRP channels, this channel was shown to participate in close regulation of calcium homeostasis in the body [4].

Increasing evidence suggests that the overexpression of TRPV6 is a common event in cancers of epithelial origin [5,6]. TRPV6 was observed to be upregulated in tissue samples originating from ovary, prostate, breast, thyroid, colon, and pancreatic tumors [7,8,9].

Prostate cancer (PCa) develops as a slow cancer in the majority of cases and is the third most lethal tumor among men [10]. It belongs to the group of malignant tumors where enhanced proliferation is accompanied by acquired resistance to apoptosis [11]. Indeed, the more PCa progresses throughout the stages, the more cells become resistant to any anticancer treatment, acquiring a more aggressive phenotype characterized by enhanced cell survival and apoptosis resistance [12]. TRPV6 is absent in healthy prostates, and it becomes expressed in prostate adenocarcinoma where the TRPV6 mRNA level has been shown to correlate significantly with the Gleason grading and is abundantly expressed in lymph node metastasis of prostate origin [7,13]. Despite the discovery of its crucial role in cancer cell proliferation and survival in vitro, no reliable tool to target the TRPV6 channel in vivo has been reported so far to be used as an effective therapy against the above cancers. Though the first efforts were already undertaken, such as peptide SOR-C13 (which also inhibits sodium channels [14]) and synthetic inhibitor TH-1177 (also inhibits the TRPV5 channel [15]), no reliable tool specifically targeting the TRPV6 channel in vivo has been reported so far to be used as efficient therapy against cancer.

The elaboration of a highly specific tool, such as an antibody, generated against the TRPV6 channel extracellular domain, would allow to specifically target and stop TRPV6-expressing cancer cells by limiting the ability of cancer cells to employ overexpressed TRPV6 channels for proliferation and viability and/or by inducing cell suppression pathways.

## 2. Materials and Methods

### 2.1. Cell Culture

Human PC3M (metastatic cell line issued from PC3 cellules grafted in vivo), PC-3, LNCaP, DU-145, and HEK293 cell lines were from American Type Culture Collection (ATCC) and were cultured in RPMI (LNCaP, PC3, and DU145), MEM (PC3M), and DMEM (HEK293) media (Gibco-BRL, CergyPontoise, France) supplemented with 10% fetal calf serum and containing kanamycin (100 µg/mL) and L-glutamine (2 mM) where necessary.

All the cells were cultured at 37 °C in a humidified atmosphere with 5% CO_2_ in air. The medium was changed three times a week and cultures were split by treating the cells with 0.25% trypsin (in PBS) for 5 min at 37 °C before reaching confluence. For the experiments, cells were seeded in 6-well plates for PCR and Western blotting and 96-well plates for the survival assays. To maintain *trpv6−/−* status of the cells, the antibiotic of selection, puromycin, at 0.1 µg/mL was added for the PC3M*^trpv6−/−^* cell line.

For the antibody treatments, the serum was decomplemented very thoroughly, i.e., heated at 60 °C for 45 min with permanent agitation, or in some cases, serum-free medium such as AIM V from Gibco™ (Billings, MT, USA) was used.

### 2.2. Electrophysiology and Solutions

Macroscopic currents were recorded from HEK-293 cells transfected with vEF1ap-5′UTR-TRPV6_CMVp-mCherry vector in the whole-cell configuration of the patch-clamp technique at room temperature using an Axopatch 200B amplifier and a Digi-data 1322A digitizer (Molecular Devices, San Jose, CA, USA). The composition of the extracellular solution for patch-clamp recording was as follows (in mM): 140 NaCl, 5 KCl, 2 CaCl_2_, 1 MgCl_2_, 10 glucose, and 10 HEPES, pH 7.4 adjusted with NaOH. The patch pipettes were filled with the intracellular pipette solution (in mM): 145 CsCl, 10 HEPES, 5 EGTA (ethylene glycol-bis(β-aminoethyl ether)-N,N,N′,N′-tetraacetic acid), and 2 MgCl_2_ (pH adjusted to 7.4 with CsOH and osmolarity 295 mOsm/kg adjusted with D-Mannitol). To detect the TRPV6 activity and unblock calcium-dependent inactivation the initial extracellular solution was replaced by the divalent-free (DVF) solution having the same ionic composition as above, but without CaCl_2_ and MgCl_2_ replaced with additional 5 mM KCl, yielding in mM: 140 NaCl, 10 KCl, 10 glucose, and 10 HEPES, pH 7.4 adjusted with NaOH. The effect of antibodies was studied by adding both rb82 and rb79 (1:500 dilution from 0.5 µg/µL) pre-diluted in DVF to the already unblocked by DVF TRPV6 channel. All chemicals were purchased from Sigma-Aldrich (St. Louis, MO, USA).

### 2.3. Calcium Imaging

Cells were plated onto glass coverslips and were loaded with 4 µM Fura-2 AM at room temperature for 45 min in the growth medium. Recordings were performed in HBSS containing the following (in mM): 140 NaCl, 5 KCl, 2 MgCl_2_, 0.3 Na_2_HPO_3_, 0.4 KH_2_PO_4_, 4 NaHCO_3_, 5 glucose, and 10 HEPES adjusted to pH 7.4 with NaOH. CaCl_2_ was adjusted to 0.07 mM or 1.8 mM depending on the experiment. The coverslips were then placed in a perfusion chamber on the stage of the microscope. Fluorescence images of the cells were recorded using a video image analysis system (Quanticell, San Francisco, CA, USA). The Fura-2 fluorescence, at the emission wavelength of 510 nm, was recorded by exciting the probe alternatively at 340 and 380 nm.

### 2.4. SDS-PAGE and Western Blotting

Semiconfluent cells were treated with an ice-cold lysis buffer containing the following: 10 mM Tris-HCl, pH 7.4, 150 mM NaCl, 10 mM MgCl_2_, 1 mM PMSF, 1% Nonidet P-40, and protease inhibitor cocktail from Sigma. The lysates were centrifuged 15,000× *g* at 4 °C for 20 min, mixed with a sample buffer containing 125 mM Tris-HCl pH 6.8, 4% SDS, 5% β-mercaptoethanol, 20% glycerol, and 0.01% bromophenol blue, and boiled for 5 min at 95 °C. Total protein samples were subjected to 8% SDS-PAGE and transferred to a nitrocellulose membrane by semi-dry Western blotting (Bio-Rad Laboratories, Hercules, CA, USA). The membrane was blocked in a 5% milk containing TNT buffer (Tris-HCl, pH 7.5, 140 mM NaCl, and 0.05% Tween 20) overnight then probed using specific rabbit polyclonal anti-TRPV6 antibodies (all at 1/500 dilution from the initial concentration of 0.5 µg/µL) and mouse monoclonal anti-β-actin (Lab Vision Co., Fremont, CA, USA, 1/1000) antibodies. Goat polyclonal anti-rabbit and anti-mouse peroxidase-conjugated secondary antibodies (Chemicon International; Temecula, CA, USA, 1/200) were used. The bands on the membrane were visualized using enhanced chemiluminescence method (Pierce Biotechnologies Inc., Escondido, CA, USA). Densitometric analysis was performed using a Bio-Rad image acquisition system (Bio-Rad Laboratories).

### 2.5. siRNA Transfection

LNCaP cells were transfected with 40 nM of siRNA against TRPV6 or siLuciferase (Eurogentec, LTD, Seraing, Belgium) using 5 µL de Lipofectamine 3000 transfection reagent (Lipofectamine 3000, Thermofisher, Waltham, MA, USA) following the manufacturer’s instructions. The siRNA sequences used to knock down the TRPV6 channel were as follows: 5′-GACUCUCUAUGACCUCACA (dTdT)-3′ and 5′-CCUGCUGCAGCAGAAGAGG (dTdT)-3′, used as a mix, and siLuciferase 5′-CUUACGCCUGAGUACUUCGA(dTdT)-3′.

### 2.6. Nucleofection

Transfection of various cell lines with different plasmids was carried out using Nucleofector (Amaxa GmbH, Köln, Germany), according to the manufacturer’s instructions. Briefly, 2 µg of the plasmid was transfected into 2 million trypsinized cells, which then were plated onto six-well dishes, 35 mm dishes, or onto the glass coverslips for 48 h.

### 2.7. Cell Survival Assay

Cell proliferation was measured using the CellTiter 96 Aqueous One Solution cell proliferation assay (Promega, Madison, WI, USA), on the basis of the cellular conversion of the colorimetric reagent MTS [3,4-(5-dimethylthiazol-2-yl)-5-(3-carboxymethoxyphenyl)-2-(4-sulfophenyl)-2H-tetrazolium salt] into soluble formazan by dehydrogenase enzymes found only in metabolically active, proliferating cells. Following each treatment, 20 μL of dye solution was added into each well in a 96-well plate and incubated for 2 h. Subsequently, absorbance was recorded at 490 nm wavelength using an ELISA plate reader (Molecular Devices, Sunnyvale, CA, USA). Cellular proliferation inhibition rate is calculated as (Acontrol − Asample)/(Acontrol − Ablank) × 100%.

### 2.8. Cell Cycle Assay

Flow cytometry assays were performed on cell populations cultured in triplicate 25 cm^2^ flasks as originally described [16]. Approximately 10^6^ cells were fixed with 1 mL ice-cold 70% methanol for 30 min. After fixing, cells were pelleted by centrifugation to remove the fixatives, washed three times with phosphate-buffered saline (PBS) at 4 °C, resuspended in 100 μL PBS, treated with 100 μL RNAse A (1 mg/mL, Sigma, St. Louis, MO, USA), and stained with propidium iodide (PI, Sigma) at a final concentration of 50 μg/mL. The stained cells were stored at 4 °C in the dark and analyzed within 2 h. The stained samples were measured on a FACScan flow cytometer (Becton–Dickinson, San Jose, CA, USA). Data were acquired for 7000 events with a variation coefficient of less than 5%, and red fluorescence was measured using a fluorescence detector 3 (FL3) on the X-axis. The data were stored and analyzed using CellQuest software to assess cell cycle distribution patterns (subG1 (apoptotic), G0/G1, S, and G2/M phases).

### 2.9. TUNEL Assay

The level of apoptosis was estimated from the number of apoptotic nuclei revealed either by TUNEL-TMR red assay (Roche Biochemicals, Basel, Switzerland, as described by manufacturer) or by Hoechst staining. The percentage of apoptotic cells was determined by counting at least five random fields for each condition performed in triplicate for each “n”.

### 2.10. Immunocytochemistry

LNCaP cells grown on glass coverslips were washed with PBS and immediately fixed in 4% paraformaldehyde in PBS. PBS-glycine (30 mM) was used to quench the reaction with no subsequent permeabilization with 0.1% Triton X-100. The cells were washed again in PBS and subjected to conventional immunostaining procedure. Alexa Fluor^®^ 488 goat anti-rabbit IgG (Molecular Probes, Eugene, OR, USA, 1/4000) was used as a secondary antibody for TRPV6 staining. Fluorescence analysis was carried out using Carl Zeiss Laser Scanning Systems LSM 510 connected to a Zeiss Axiovert 200 M with 40X1.4 numerical aperture oil immersion lens at room temperature. Both channels were excited, collected separately, and then merged using software Carl Zeiss LSM Image Examiner (3.1.0.99).

### 2.11. Plasmids

The whole TRPV6 cDNA containing 5′-UTR on the pCAGGS vector was provided by Dr. Ulrich Wissenbach from Universität des Saarlandes, Germany. This sequence was used to obtain a final vEF1ap-5′UTR-TRPV6_CMVp-mCherry vector (E-Zyvec, Loos, France) which was nucleofected into the HEK cells, and the transfection rate was evaluated using a control vEF1ap-5′UTR_CMVp-mCherry vector [16].

### 2.12. Antibody Production

The 15 amino acid epitopes were coupled to a KLH protein to its N-terminus and injected into the rabbits once per week during four weeks following by the final bleed (Eurogentec, LTD) as described previously [17]. The serum was tested in ELISA using antigen-coated plates followed by affinity purification in columns against the same bound antigen. Finally, the affinity-purified antibodies were supplied, diluted 50/50 *v*/*v* with the glycerol, and stored at −20 °C.

### 2.13. Reagents

All reagents were purchased from Sigma (Sigma, L’Isle d’Abeau Chesnes, France) unless otherwise specified.

### 2.14. Data Analysis

For each type of experiment the data were accumulated from at least three measurements (n). A big N indicates the number of cells used in the statistical analysis. Data were analyzed using Origin 7.0 (Microcal Software Inc., Northampton, MA, USA) software. Results were expressed as Mean ± S.E.M. and Mean ± S.E.D. for the calcium imaging and patch-clamp. ANOVA was used for statistical comparison of the differences and *p* < 0.05 was considered significant. In the graphs, (*), (**), and (***) denote statistically significant differences with *p* < 0.05, *p* < 0.01, and *p* < 0.001, respectively.

## 3. Results

### 3.1. Design and Validation of Antibodies Raised against Extracellular Epitopes of the TRPV6 Protein

**Figure 1:**
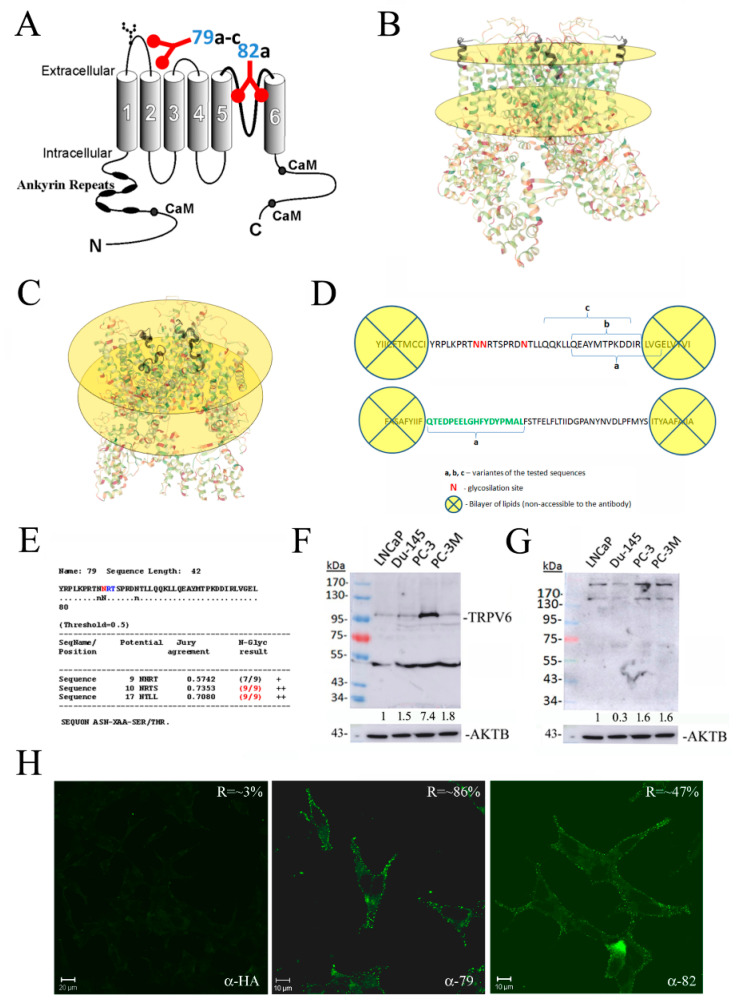
Design and validation of two rabbit polyclonal anti-TRPV6 channel antibodies. (**A**) The general scheme of the channel and the relative position of the epitopes for the two antibodies, rb79a-c and rb82 (base image of the channel from http://atlasgeneticsoncology.org/, accessed on the 1 May 2009). (**B**,**C**) A 3D image of the TRPV6 channel generated using PDB database (https://www.rcsb.org/, acessed on the 9 September 2016; 6BO8: Cryo-EM structure of human TRPV6 in nanodiscs) with the lipid bilayer limits (interior and exterior) shown as yellow circles where the rb79a (**B**) or rb82 (**C**) epitopes on the each channel tetramer are highlighted in black color. (**D**) Schematic diagram with the amino acid positions within S1 and S2 transmembrane domains (upper sequence) and amino acid positions within S5 and S6 transmembrane domains (lower sequence), epitope variants (a–c), and glycosylation sites (N). (**E**) Analysis of probability of N-glycosylation using NetNGlyc 1.0 software. (**F**) The representative immunoblotting of the total lysates of the LNCaP, DU-145, PC-3, and PC-3M cells revealed with rabbit polyclonal anti-TRPV6 antibody rb79a. (**G**) The representative immunoblotting of the total lysates of the LNCaP, DU-145, PC-3, and PC-3M cells revealed with rabbit polyclonal anti-TRPV6 antibody rb82. The same AKTB housekeeping gene has been used for quantifications, n = 1. (**H**) The immunofluorescence experience of the LNCaP cells pretreated with the rabbit polyclonal anti-HA and anti-TRPV6 antibodies rb79a and rb82, then fixed with 3.5% of paraformaldehyde without permeabilization and revealed with the anti-TRPV6 antibody rb79a. The ratio indicated in the right upper corner shows the specificity of the staining signal as compared to the adjacent background.

Four polyclonal antibodies were raised against the extracellular part of the TRPV6 channel (79a-c and 82). The first loop between S1 and S2 transmembrane domains and a pore region between S5 and S6 were considered (Figure 1A). For the design of peptide sequences, the position relative to the plasma membrane, lipid bilayer, glycosylation sites, as well as the already available 3D structure of the TRPV6 channel were considered [18] (Figure 1B,C). A 3D image of the TRPV6 channel was generated using the PDB database (https://www.rcsb.org/; accessed on 09 September 2016, 6BO8: Cryo-EM structure of human TRPV6 in nanodiscs). A total of 37 amino acids span the first extracellular loop situated between S1 and S2 transmembrane domains and 44 amino acids span the third extracellular loop situated between S5 and S6 transmembrane domains (Figure 1D). Of them, three residues are those of Asparagin, N. A detailed analysis using NetNGlyc 1.0 software demonstrated the most probable second and third sites of N-glycosylation, RTNNRT, and RDNTL (Figure 1E). The presence of these sites and the N-glycosylation thereof will deny the potential steric access to the epitope by the antibody. From the other side, the lipid bilayer will preclude the antibody from binding to the respective amino acids of the epitope.

The immunoblotting of the total lysates of the prostate cancer cell lines LNCaP, DU-145, PC-3, and PC-3M cells revealed that using rabbit polyclonal anti-TRPV6 antibody rb79 showed an expected size of the glycosylated form of the protein around 95–100 kDa. In addition, the unglycosylated sequence was observed at around 84 kDa, as well as a band of around 50 kDa, which was consequently turned to be unspecific, being often present in the ladder lane (Figure 1F) [17]. From those of the 79 epitope, only rb79a as the best antibody matching the right size was retained for the further experiments (designated further as rb79), whilst other variants, rb79b and rb79c, were eliminated (Supplementary Figure S1A,B). As for the 82 epitope, the results were not satisfying (showing probably TRPV6 dimers and tetramers), but since it was the only antibody available, it was retained for further experiments (designated rb82) (Figure 1G).

Since the generated antibodies were supposed to act extracellularly, an immuno-fluorescence experiment (confocal microscopy) without cell permeabilization and 15 min of preincubation using either rb79 or rb82 followed by the 3.5% PFA fixation was carried out (Figure 1H). A rabbit polyclonal antibody of the same isotype (IgG1), the anti-HA epitope, was used as a control. The data clearly show the specific membrane binding for the antibody rb79 on the plasma membrane. Since the staining was less pronounced for the antibody rb82, a Carl Zeiss LSM Image Examiner analysis of the pixel intensities in the selected membrane area was employed. The ratio indicated in the right upper corner shows the specificity of the staining signal as compared to the adjacent background (Figure 1H). The intensity of staining was statistically significantly higher for the antibody rb82 as compared to the control antibody anti-HA, but still twice lower than that of rb79. In addition, the control co-staining with the plasma membrane marker such as cholera toxin (CTX), marking G2M lipids, was performed to prove a plasma membrane localization of the TRPV6 channel (Supplementary Figure S1C).

Thus, these two anti-TRPV6 antibodies, rb79 (i.e., rb79a) and 82, were chosen to be used in further functional experiments.

### 3.2. Antibody Treatments Increase Store-Operated Capacitive Calcium Entry in PCa Cells

**Figure 2:**
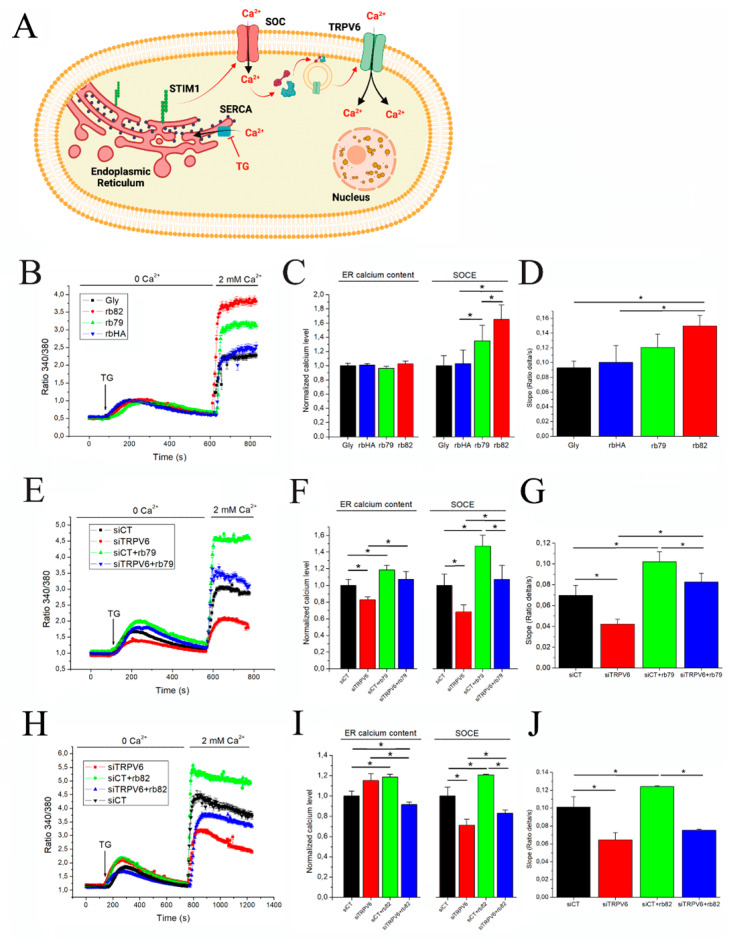
Effects of the antibody treatments on the store-operated calcium entry, ER calcium content, and SOCE slope. (**A**) Schematic diagram of the store-operated capacitive calcium entry (SOCE) where TRPV6 channel takes an important part. The inhibition of the SERCA pump with Thapsigargin provokes calcium leak, which will empty calcium stores and thus activate store-operated channels (SOC), which, in turn, will activate TRPV6 channel, taking an important part in the amplification of the calcium entry inside the cells (created using source BioRender.com, accessed on 30 September 2022). (**B**) The SOCE in the LNCaP cells pretreated 5 min with either glycerol (Gly) or rabbit polyclonal anti-HA or anti-TRPV6 antibodies, rb79 and rb82. (**C**) A corresponding quantitative representation of the SOCE and ER calcium content (calculated as a maximum amplitude) affected by antibody-induced treatments shown in (**B**); n = 3, * *p* < 0.05. (**D**) A slope of SOCE calculated as a ratio delta/sec for each condition; n = 3, * *p* < 0.05. (**E**) SOCE into the LNCaP cells under the anti-TRPV6 antibody rb79 pretreatment of 5 min with the knockdown of TRPV6 channel (siRNA, 40 nM, 48 h). (**F**) A corresponding quantitative representation of the SOCE and ER calcium content affected by the treatments shown in (**E**); n = 3, * *p* < 0.05. (**G**) A slope of SOCE calculated as a ratio delta/sec for each condition; n = 3, * *p* < 0.05. (**H**) SOCE into the LNCaP cells under the anti-TRPV6 antibody rb82 pretreatment of 5 min with the knockdown of TRPV6 channel (siRNA, 40 nM, 48 h). (**I**) A corresponding quantitative representation of the SOCE and ER calcium content affected by the treatments shown in (**H**); n = 3, * *p* < 0.05. (**J**) A slope of SOCE calculated as a ratio delta/sec for each condition; n = 3, * *p* < 0.05.

TRPV6 was shown as an important element of store-operated calcium entry (SOCE) into the PCa cells allowing the use of this mechanism to detect and analyze TRPV6 activity [19]. This mechanism is triggered by the emptying of calcium stores in the endoplasmic reticulum (ER). Inhibition of the SERCA pump with Thapsigargin (1 µM) is used to induce a calcium leak, which will empty calcium stores and thus activate store-operated (SOC) channels such as Orai1, TRPC1, etc. Their activation will induce calcium entry, translocating TRPV6 channels at the plasma membrane and thus amplifying SOCE [19] (Figure 2A).

The preincubation of PCa cells such as LNCaP for 5 min with either glycerol (Gly) or rabbit polyclonal anti-HA or anti-TRPV6 antibodies rb79 and rb82 (all normalized at 1/500 dilutions of the stock 0.5 µg/µL) led to pronounced effects, such as a selective and significant increase in the SOCE levels with no noticeable change in ER calcium content level calculated as a maximum amplitude (Figure 2B,C). In addition, the slope of the SOCE curve was significantly higher for the rb82 condition as compared to both glycerol and rbHA (Figure 2D). To show that these effects are mediated via the TRPV6 channel, both antibodies were subjected to an additional control using the siRNA strategy for the TRPV6 knockdown. The SOCE was significantly decreased while knocking down the TRPV6 channel, whereas the antibody rb79-mediated increase in SOCE (siCT+rb79) was significantly attenuated when the siTRPV6 strategy was used (Figure 2E,F). Moreover, significant ER calcium content changes were noticed in siTRPV6 and siCT+rb79 conditions similar to the SOCE amplitude (Figure 2F). As for the slope of the SOCE, the rb79 antibody significantly increased it, being attenuated by siTRPV6 (Figure 2G).

The same data were obtained using the pretreatment with the rb82 antibody, which was able to significantly increase SOCE and ER calcium content (Figure 2H,I). Similarly, siRNA against the TRPV6 channel was capable of significantly decreasing both the rb82-induced SOCE and ER calcium content, though it failed to decrease the ER calcium content by itself (Figure 2H,I). As for the SOCE slope, rb82 significantly increased it with the consequent decrease by siTRPV6, suggesting the implication of TRPV6 therein (Figure 2J).

Thus, both antibodies, rb79 and rb82, amplify SOCE and increase ER calcium content and speed of SOCE while a greater amount of calcium enter inside cells via TRPV6 channels.

### 3.3. Antibodies 79 and 82 Directly Affect TRPV6-Induced Currents

**Figure 3:**
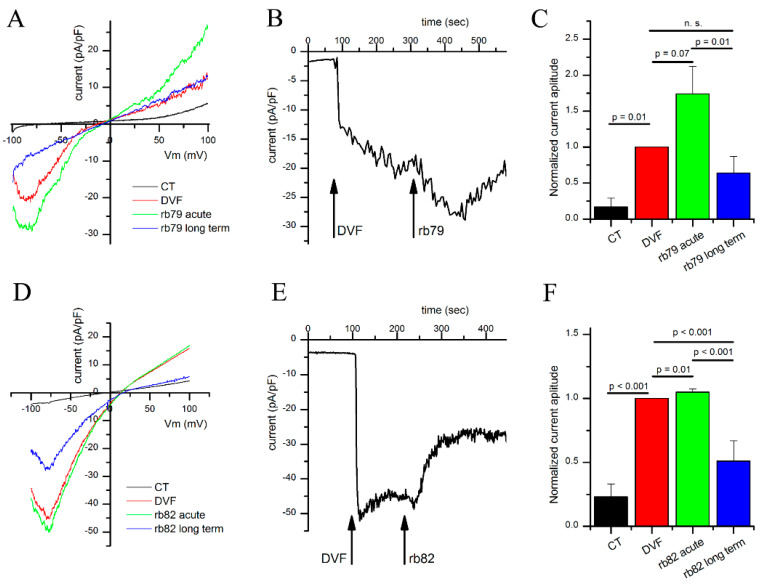
The rb79 and rb82 antibodies directly affect the whole-cell TRPV6 currents. Panels (**A**,**D**) show representative IV curves evoked by the −100/+100 mV voltage ramp are shown for the HEK cell transfected with the vEF1ap-5’UTR-TRPV6wt_CMVp-mCherry vector. Curves show whole-cell TRPV6 currents in either base HBSS medium (black), DVF medium alone (red), or DVF medium containing 1:500 dilution of the rabbit polyclonal anti-TRPV6 antibody rb79 (**A**) or rb82 (**D**), during the acute phase (green) or long-term inhibitory phase (blue). (**B**,**E**) A representative time-course of the amplitudes of the inward TRPV6 whole-cell currents following solution changes and application of the rb79 (**B**) or rb82 (**E**) antibodies, as indicated by arrows. Note a transient increase in the TRPV6 current immediately after application of the antibody, followed by the sustained inhibition phase. (**C**,**F**) Bar plots summarizing average whole-cell currents under indicated conditions for rb79 ((**C**), n = 4) or rb82 ((**F**), n = 12); *p*-values indicated for the corresponding pairs of conditions on the top of the horizontal lines.

A gold standard in extracellular antibody action on the ion channel is the technique of patch-clamp, allowing to measure the ion currents passing through the particular channel since each of them has a unique conducting feature or signature. The specificity of the developed antibodies rb79 and rb82 was verified by measuring their effect upon TRPV6-specific whole-cell currents recorded from the HEK cell transfected with vEF1ap-5′UTR-TRPV6wt_CMVp-mCherry (Figure 3). Cells were initially incubated in the physiological solution HBSS containing 2 mM Ca^2+^, known to block TRPV6 activity because of the Ca^2+^-calmodulin interactions with TRPV6 [20,21,22]. TRPV6-specific currents were recorded by changing the extracellular (bath) solution by the divalent-cation-free (DVF) solution, commonly known to prevent Ca^2+^-induced TRPV6 inactivation [23] (Figure 3A,B,D,E). Then, the antibodies prediluted in the DVF solution were added to the bath to study their effects. For both antibodies, a transitory activation of TRPV6 currents occurred (much shorter for rb82), representing an acute effect of the antibody. This transient activation was followed by an inhibition in TRPV6 activity (much pronounced for rb82). The rabbit polyclonal anti-TRPV6 antibody rb79 (1/500 of 0.5 µg/µL) exhibited a significant increase in the TRPV6 current during the acute phase (Figure 3A–C), while the rb82 antibody produced only a short though significant stimulation in the acute phase (Figure 3D–F). After this transient stimulatory phase, both antibodies inhibited TRPV6 currents by a long-term inhibition of approximately 50%. Thus, the mechanism of action of both antibodies observed using patch-clamp whole-cell configuration seems to be biphasic and complex, involving the transient increase in amplitude followed by the progressive decline.

### 3.4. Decrease in Cell Survival via TRPV6 Channel Activation

**Figure 4:**
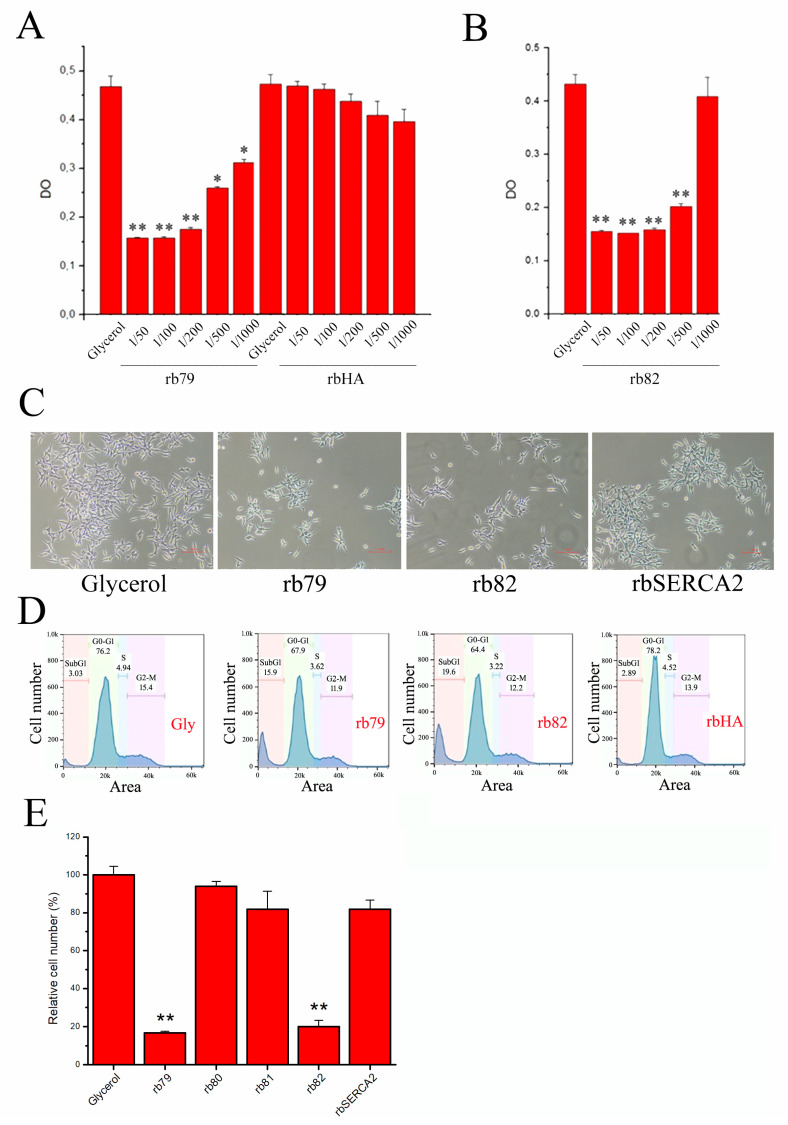
TRPV6 modulation using rb79 and rb82 decreases cell survival. (**A**) Cell survival assay (MTS) of LNCaP cells treated either with the equivalent quantity of the glycerol or different antibody (anti-TRPV6 antibody rb79 and anti-HA of the same isotype for 3 days) dilutions normalized to the initial quantity of 0.5 µg/µL; n = 3, * *p* < 0.05, ** *p* < 0.01. (**B**) The same cell survival of LNCaP cells treated with anti-TRPV6 antibody rb82. (**C**) Photos of the LNCaP cells treated for 3 days with the 1/500 dilution of above experiment with either anti-TRPV6 antibodies rb79 or rb82 or anti-SERCA2B of the same isotype in addition to the glycerol. (**D**) Cell cycle assay with a distinct sub-G1 peak of the LNCaP cells using anti-TRPV6 antibodies rb79 and rb82 and anti-HA of the same isotype (at 2.5 ng/mL) as compared to the glycerol for 3 days. (**E**) Cell count of the LNCaP cells treated for 3 days with anti-TRPV6 antibodies rb79 and rb82 as well as rb80 and rb81 (raised against intracellular epitopes of the TRPV6 channel, N and C terminus, correspondingly), and anti-SERCA2B of the same isotype as rb79 and rb82 and glycerol; n = 3, ** *p* < 0.01. All antibodies were used at the same concentration of 2.5 ng/mL.

As soon as we proved the direct action of our antibodies on the TRPV6 channel, the crucial question was whether these antibodies, i.e., the rabbit polyclonal anti-TRPV6 antibodies rb79 and rb82, are capable of influencing PCa cell survival in vitro while activating the TRPV6 channel. For that, LNCaP cells were incubated for 72 h with either glycerol or different dilutions of anti-TRPV6 antibodies rb79 and rb82 (Figure 4A,B, respectively). Moreover, a control antibody anti-HA was used in different dilutions, showing no difference as compared to control (glycerol). Both rb79 and rb82 were capable of significantly decreasing the cancer cell survival rate (Figure 4A,B).

Since the cell survival assay is based on evaluating the cytochrome p-450 activity and therefore is a complex assay measuring both cell proliferation and cell death, a panel of additional techniques was used. In our cell count assay, we photographed the cell density prior to cell count (Figure 4C), showing clearly different cell density in the rb79- and rb82-treated dishes as compared to the control rabbit antibody of the same isotype, anti-SERCA2B (anti-HA or rbHA had the same zero effect, data not shown). This different control antibody, rabbit polyclonal anti-SERCA2B, was used to highlight the specificity of both rb79 and rb82 antibodies.

Further, the cell cycle assay was performed showing that the subG1 peak rather than different phases of the LNCaP cell cycle was affected. Treatment using antibodies rb79 and rb82 for 72 h has shown a significant effect on the subG1 peak as compared to both glycerol and rbSERCA2B of the same isotype, suggesting that anti-TRPV6 antibodies act by inducing apoptosis rather than decreasing the proliferation (Figure 4D).

Cell counts of the rb79- and rb82-treated prostate cancer cells are shown in Figure 4E, where a panel of additional antibodies was tested. In fact, two more control antibodies were used, i.e., those which target the TRPV6 channel from inside of the cell and therefore cannot target TRPV6 channel present on the intact living cells (rb81—C-terminus, rb80—N-terminus: both intracellular). All these antibodies in addition to the glycerol and a control anti-SERCA2B antibody were inefficient in affecting LNCaP cell growth. Our data strongly suggest the critical involvement of the TRPV6 channel in PCa cell survival, and thus the modulation of its activity may be extremely beneficial for the treatment of PCa.

Moreover, in our pilot experiment we tested the effects of both rb79 and rb82 antibodies (as compared to another control antibody of the same isotype: rabbit polyclonal anti-GFP) on a PC3M*^trpv6−/−^* and PC3M*^trpv6+/+^* prostate cancer hormone-refractory cell line (Supplementary Figure S1E). No effect of either rb79 or rb82 was observed in the PC3M*^trpv6−/−^* cell line. These data confirmed the specific targeting of the TRPV6 channel by both rb79 and rb82 antibodies.

**Figure 5:**
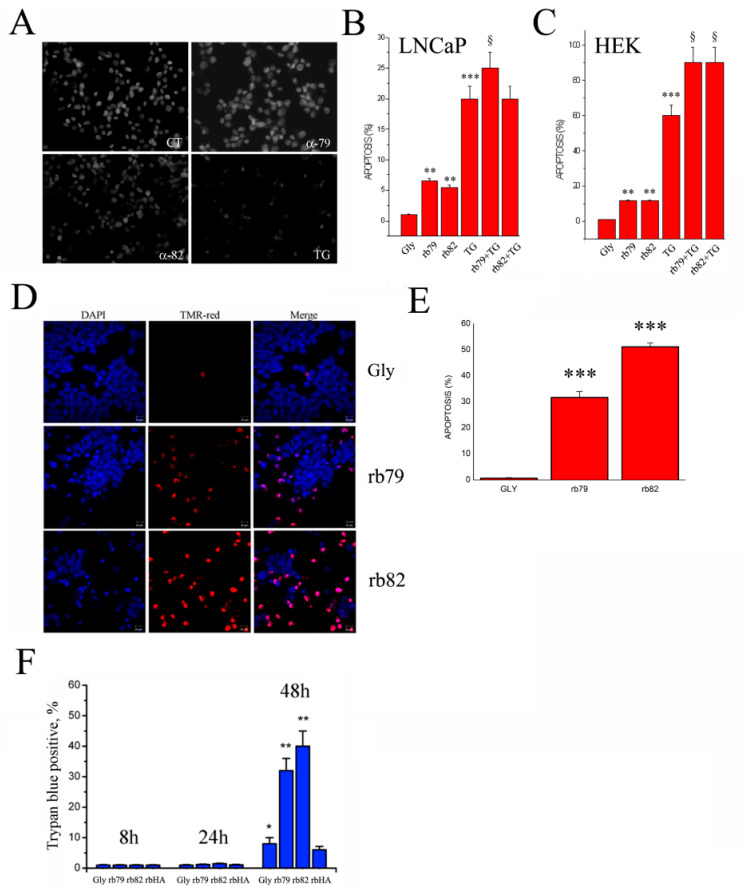
Activation of the TRPV6 channel using antibodies rb79 and rb82 induces apoptosis of prostate cancer cells. (**A**) Apoptosis assay using Hoechst staining of LNCaP cells. Cells were pretreated for 72 h either with the equivalent quantity of the glycerol (CT) or anti-TRPV6 antibodies rb79 and rb82 for 72 h (1/500 of 0.5 µg/µL). Treatment with 1 µM of the Thapsigargin (TG) for 72 h was used as a positive control to induce apoptosis. (**B**) Quantification of the apoptosis rate using Hoechst staining of LNCaP cells; n = 3, ** *p* < 0.01, *** *p* < 0.001, § *p* < 0.05 as compared to TG-only (1 µM, 72 h) treatment. (**C**) Quantification of the apoptosis rate using Hoechst staining of HEK TRPV6-expressing cells; n = 3, ** *p* < 0.01, *** *p* < 0.001, § *p* < 0.05 as compared to TG-only (1 µM, 72 h) treatment. (**D**) Apoptosis assay using TMR-red TUNEL assay of LNCaP cells treated with anti-TRPV6 antibodies rb79 and rb82 at 2.5 ng/mL and the glycerol (Gly); n = 3. (**E**) Statistical data analysis of the number of apoptotic cells observed in (**D**); n = 3, *** *p* < 0.001. (**F**) Trypan blue staining of the LNCaP cells treated with the equivalent quantity of the glycerol (Gly) or anti-TRPV6 antibodies rb79 and rb82 for 72 h (at 2.5 ng/mL each), or anti-HA of the same isotype, carried out during 8, 24, and 48 h; n = 3, * *p* < 0.05, ** *p* < 0.01.

Further, a classical apoptosis assay, Hoechst staining, was used to confirm the hypothesis of the apoptosis induction (in addition to the sub-G1 peak, Figure 4D) by the anti-TRPV6 antibodies rb79 and rb82 (Figure 5A). Though this technique is limited to the late-phase apoptosis, it permitted to see the significant increase in the number of apoptotic cells treated with both rb79 and rb82. Thapsigargin 1 µM for 3 days was used as a positive control since it induces calcium-dependent apoptosis via unfolded protein response in the long-term treatment [24]. Quantification of the apoptotic cells showed a significant death rate induced during 3 days of treatment with the anti-TRPV6 antibodies rb79 and rb82, not only in the PCa cell line LNCaP (Figure 5B) but also in non-cancerous HEK cells (Figure 5C), which are much more apoptosis-sensitive as compared to PCa cells and express TRPV6 (Supplementary Figure S1D). Further, the treatment of LNCaP cells with rb79 and HEK with both rb79 and rb82 cells together with TG potentiated the effects of calcium-induced apoptosis by TG.

The other reliable and more sensitive apoptosis assay, TMR-red TUNEL, was performed to confirm the data, and showed a much higher rate of the apoptosis in rb79- and rb82-treated conditions as compared to the glycerol treatment (Figure 5D). The apoptosis rate increased by 31.6 ± 2.3% in rb79-treated conditions and by 51.1 ± 1.5 in rb82 conditions (Figure 5E).

Finally, to exclude necrosis as a possible mechanism, a time series of 8, 24, and 48 h was performed using a trypan blue staining, showing the late appearance of the stained cells as an indicator of middle to late apoptosis where the membrane integrity is compromised (Figure 5F).

Thus, both rb79 and rb82 antibodies induce cell death via apoptosis.

### 3.5. Plasma Membrane TRPV6 Expression Determinates Cancer Cell Fate

**Figure 6:**
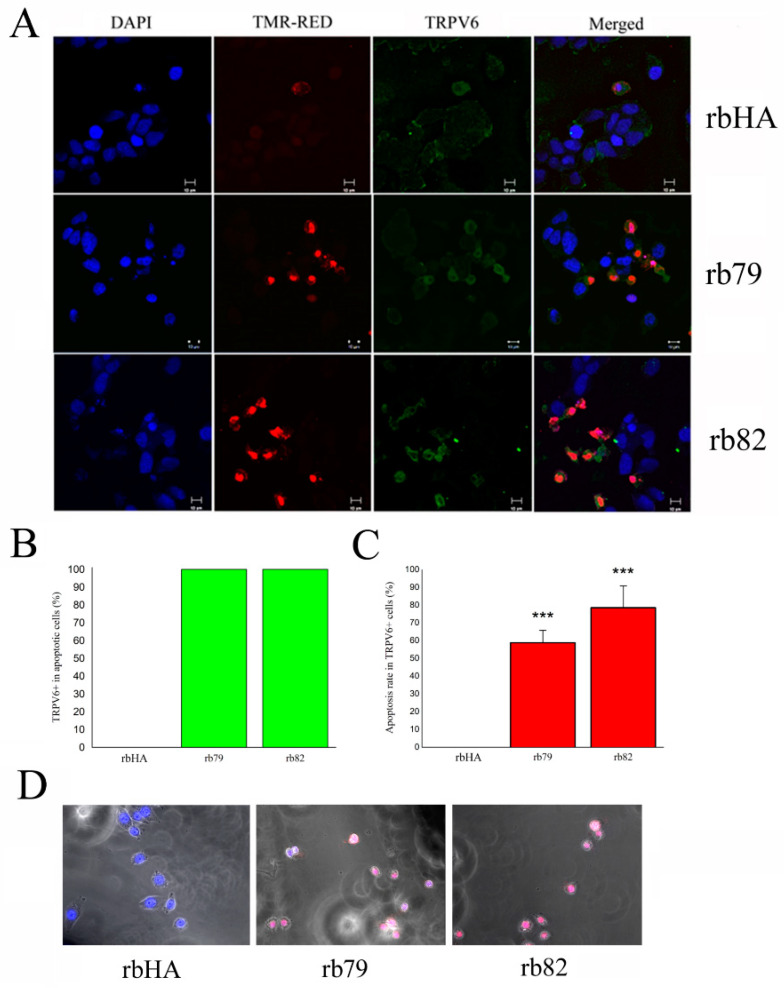
TRPV6 expression on the plasma membrane is required for the apoptosis induction of TRPV6-expressing cancer cells. (**A**) Apoptosis assay using TMR-red TUNEL assay of LNCaP cells pretreated with anti-TRPV6 antibodies rb79 and rb82 for 72 h (at 2.5 ng/mL each) or anti-HA of the same isotype. TRPV6 channel was revealed using anti-TRPV6 antibody rb79. No plasma membrane permeabilization was used. (**B**) Number of TRPV6+ cells (membrane staining) in apoptotic cells following rb79 and rb82 treatments, n = 3. (**C**) Apoptosis rate in TRPV6+ cells (membrane staining) following rb79 and rb82 treatments, n = 3, *** *p* < 0.001. (**D**) Video microscopy of LNCaP cells treated with the antibodies rb79 and rb82 and anti-HA antibody for 72 h (all at 1/500 of 0.5 µg/µL), co-stained with the propidium iodide (see Supplementary Movies S1–S3).

Our data show that both antibodies activating the TRPV6 channel, rb79 and rb82, are not 100% effective in the cell death induction of PCa cells, and thus confirm the hypothesis that only cells which are void of TRPV6 expression will survive the treatment. For that, the co-staining of apoptotic cells treated during 3 days with anti-TRPV6 antibodies rb79 and rb82 the and anti-HA antibody was performed. In this protocol, cells were first fixed and then pretreated without permeabilization with the antibody rb79 to detect the TRPV6 channel on the plasma membrane followed by the TMR-red TUNEL procedure (Figure 6A). Our data clearly show that 100% of apoptotic LNCaP cells where apoptosis was induced either by rb79 or rb82 expressed TRPV6 on the plasma membrane (Figure 6B). From the other side, not all the TRPV6 positive on the plasma membrane cells was subjected to the apoptosis induced either by rb79 (58.9 ± 7) or rb82 (78.6 ± 12) during the 72 h of treatment (Figure 6C).

Finally, our PCa cells were subjected to the video microscopy experiment where the cell behavior was recorded for 3 days treated with antibodies rb79 and rb82 and anti-HA antibodies (all at 1/500 of 0.5 µg/µL), co-stained with the propidium iodide to monitor for cell integrity (Figure 6D: see Supplementary Movies S1–S3). As it can be seen from the movies, both anti-TRPV6 antibodies rb79 and rb82 are potent cell death inducers for the majority of the PCa cells.

These data suggest that the effects of rb79 and rb82 depend on the presence of TRPV6 at the plasma membrane, and thus, only cancer cells which do not express the TRPV6 channel on the plasma membrane will survive.

## 4. Discussion

In the current work, we have undertaken an effort to create a proof of concept for the targeting of the TRPV6 calcium channel in prostate cancer. Though the first efforts were already undertaken, such as peptide SOR-C13 (which inhibits also sodium channels [14]) and synthetic inhibitor TH-1177 (also inhibits the TRPV5 channel [15]), no reliable tool specifically targeting the TRPV6 channel in vivo has been reported so far to be used as an efficient therapy against cancer. Our proof of concept for the targeting of the TRPV6 calcium channel in prostate cancer is based on the generation of rabbit polyclonal antibodies capable of recognizing and binding to the extracellular epitopes of the channel, such as the first extracellular loop between S1 and S2 for rb79 and the pore region for rb82. Though the first data on the use of both rb79 and rb82 in the TRPV6 detection in vitro have already been published [17], only rb79 was retained for such a purpose, whereas rb82 did not show any expected TRPV6 monomer size detection. However, in all in vitro applications where TRPV6 stayed in its native 3D conformation, rb82 did work, and thus was tested in TRPV6 targeting of living cancer cells. It should also be noted that rabbit polyclonal antibodies should not be underestimated even if they represent a pool of antibodies against one particular epitope. Rabbit species are more distant to humans in the evolutional aspect, as are mouse species widely used for the monoclonal antibody generation and further antibody humanization. Thus, the rabbit host represents higher chances to develop a prospective antibody from the point of view of antigenicity divergence and thus immunogenicity for its body as compared to a mouse host.

In our experiments, we generated two antibodies capable of recognizing TRPV6 extracellular domains, binding to them, altering protein conformation in the lipid bilayer, and thus changing its permeability. Indeed, antibodies, as protein entities, are known to be capable of changing protein conformation upon binding. A large amount of data exist showing that binding of IgGs to the protein can modify the conformation/structure of a protein [25,26,27]. Since an ion channel’s function depends directly on its spatial conformation, its interaction/insertion into lipid bilayer, its thermodynamic state, mono/tetrameric structure, and many more parameters/criteria, any binding to the protein channel in its natural, functional conformation will definitely induce a switch in ion channel conformation and thus activity [27,28,29]. Knowing that even one ion can change ion channel permeability, binding of an ≈150 kDa entity such as IgG will definitely provoke a pore conduction shift and thus decrease or increase the ion current.

As for rb79 and rb82, their binding to TRPV6 led to the transient activation of the latter while activating calcium currents and increasing capacitive calcium entry—deleterious for a cancer cell by inducing its death [30,31,32]. In our SOCE experiments using siRNA, we could not completely ablate the effects of TRPV6 activation, since the efficiency of siRNA is not absolute [16,19] and enough protein still remains to partially sustain the observed effects. Moreover, this residual amount of TRPV6 channels present on the plasma membrane after siRNA treatment was enough to amplify SOCE effects upon rb79/rb82 binding. It should be noted that the increases in SOCE slopes are consistent with the TRPV6 activation by rb79/rb82, though the ER calcium content was less reliable because of the short-term preincubation by both rb79 and rb82 antibodies and was out of the scope of this study.

In the case of rb79, the activation of TRPV6 currents in HEK293 cells overexpressing the TRPV6 channel was deeper and more pronounced than those of rb82, which were transient. It is likely that the mechanism of action of the rb82 antibody is more complex than just transient activation. In fact, the initial transient increase in the TRPV6 activity under rb82 action, which is still statistically significant, was followed by a sustained decrease in its activity, which may have further consequences on the TRPV6 activity in cancer cells. However, in our LNCaP cells, no decrease in SOCE upon neither rb82 nor rb79 treatments was observed. The high selectivity of the TRPV6 channel and its strong current provided during the initial phase of SOCE upon rb79 and rb82 binding would explain this sustained increase in SOCE, even when the phase of decrease in TRPV6 activity would follow.

On the other hand, the measurement of SOCE is not a pure test of TRPV6 activity by itself because of the many other SOCE components such as Orai1, TRPC1, etc., however, it is still very pertinent for the functional activity of TRPV6 in its contribution to SOCE, i.e., its amplification [19]. In addition, TRPV6 is prone to translocation from the periplasmic vesicles upon stimulation of SOCE and this fact should be taken into account, since an antibody can potentially stabilize/retain TRPV6 channels on the plasma membrane while strengthening Ca^2+^ overload and apoptosis induction even if long-term inhibition by rb82 occurs. Thus, the mechanism of action of both antibodies seems to be biphasic and complex and will be a matter of the future studies, whereas in this paper we focus on the transient activation effects leading to the increase in functional SOCE with its consequence to the cancer cells.

The activation of calcium channels, including the TRP channel superfamily, would lead to the increase in local calcium. This increase will involve calcium-dependent factors capable of inducing cell death, which include calcium-dependent cysteine proteases such as calpains and the calcium-dependent serine-threonine phosphatases such as calcineurin, that form a combined calpain–calcineurin signaling complex sensitive to local calcium raises [33]. As an example, an activated TRPC5 current was shown to induce a sustained increase in cytosolic calcium, which would lead to cell damage via the CaMKII and calpain–caspase-dependent pathways [34]. The stimulation of the TRPM2 channel was shown to make cells be more sensitive to apoptosis via enhanced ROS levels and calpain activity, and thus the induction of caspase 3 activity triggering the downstream apoptotic pathway [35]. A similar mechanism has been published for the activation of the TRPM8 channel by menthol, the specific agonist of TRPM8, which induced a sustained increase in cytosolic calcium concentration and production of intracellular reactive oxygen species and reduced the cell numbers and survival of prostate cancer cells [36] and human melanoma [37]. Future investigations on the precise intracellular mechanisms where TRPV6 activation by rb79 and rb82 would induce massive calcium entry and calcium-dependent apoptosis induction are needed.

TRPV6 is a highly calcium-selective channel [3,4], and thus its targeting may have a deleterious effect on the cancer cell. The examples of calcium channel targeting antibodies in cancer are as follows: polyclonal antibody that targets a non-functional form of P2X7 (nfP2X7) for the treatment of basal cell carcinoma [38]; an inhibiting mouse monoclonal antibody against isoform 5 of the α2δ1 subunit of voltage-gated calcium channels to target hepatocellular carcinoma [39]; and an inhibiting mouse monoclonal anti-Cx43 E2 antibody (connexon), which suppresses Cx43 docking to target breast cancer [40].

We therefore can hypothesize that when rb79 and rb82 bind to this channel, it is not naturally regulated anymore (there is no calcium–calmodulin inactivation, because the channel is in a tense conformation), and thus the calcium entry via TRPV6 becomes uncontrollable. This uncontrolled calcium entry will finally lead to the apoptosis, as we show in this work. Our data clearly show that all the apoptotic cells expressing the TRPV6 channel on the plasma membrane were dead upon the action of both rb79 and rb82. These data allow to suggest that the activation of such a highly calcium-selective channel may be more efficient than ordinary inhibition, since the massive or uncontrolled calcium entry has been shown to be lethal to a cell [19,24,41].

Finally, our work demonstrates the use of the rabbit polyclonal antibodies, which are by their virtue transiently activating antibodies and have a very high if not absolute efficiency against prostate cancer cells expressing TRPV6 on the plasma membrane. In addition, being polyclonal they are potentially limited to topical use. The generation of the rabbit monoclonal antibodies or mouse monoclonal antibodies having similar virtues or against the same epitopes with the subsequent humanization and use in clinics would be of prime importance.

## 5. Conclusions

We generated two antibodies against extracellular epitopes of the highly calcium-selective channel TRPV6. These antibodies were shown to be able to transiently activate TRPV6 channels and thereby amplify store-operated calcium entry. This transient activation of TRPV6 and increase in intracellular calcium led to the apoptosis induction in prostate cancer cells with the subsequent death of all cells, which expressed TRPV6 on the plasma membrane. Thus, activation of the TRPV6 channel *per se* is extremely efficient and is likely to be more prospective than its inhibition.

## 6. Patents

This work is an integral part of the patent EPO, number EP21306438. Title: “ANTIBODIES AGAINST EXTRACELLULAR EPITOPES OF HUMAN TRPV6 CHANNEL AND THEIR DIAGNOSTIC AND THERAPEUTIC USES”, inventors: Dr. V’yacheslav LEHEN’KYI, Dr. Aurélien HAUSTRATE, and Prof. Natalia PREVARSKAYA, filed on 14 October 2021. (Submission number 1000504057; application number EP21306438.9; no. to be used for priority declarations EP21306438; date of receipt 14 October 2021.)

## Data Availability

Not applicable.

## Supplementary Materials

The following supporting information can be downloaded at: https://www.mdpi.com/article/10.3390/cancers15061825/s1. Figure S1: Supplementary data; Videos: The effects of antibodies on LNCaP cell viability. Video microscopy of LNCaP cells treated with the antibodies rb79 (S2) and rb82 (S3) and anti-HA antibody (S1) for 72 h (all at 1/500 of 0.5 µg/µL), co-stained with propidium iodide.
