# Supplementary figures and images for: TRPV6 Calcium Channel Targeting by Antibodies Raised against Extracellular Epitopes Induces Prostate Cancer Cell Apoptosis
Source: Cancers (Basel). 2023 Mar 17;15(6):1825. doi: 10.3390/cancers15061825 (PMC10046753; doi:10.3390/cancers15061825)

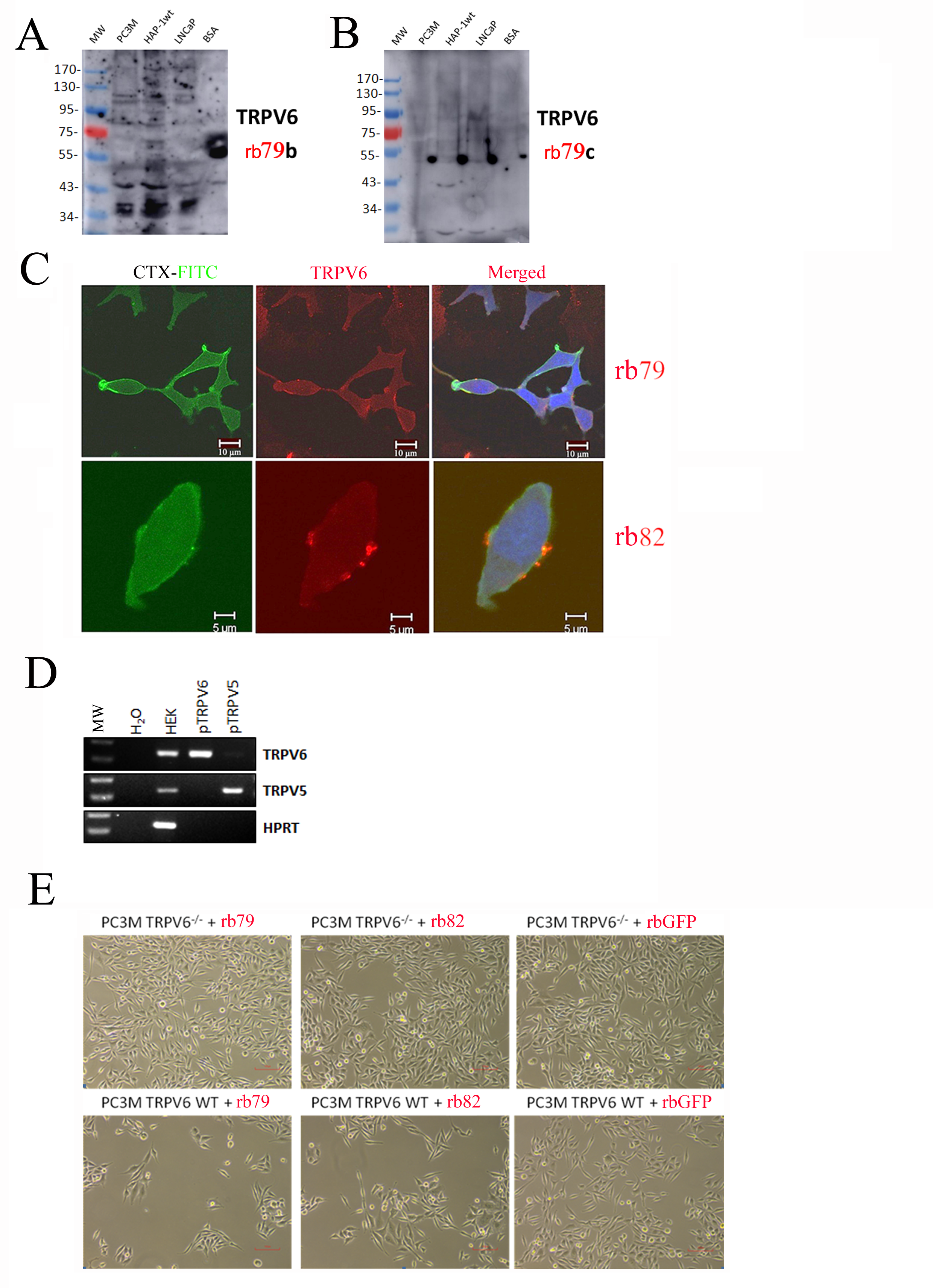

Supplement: Supplementary file 1 [file cancers-15-01825-s001.zip › Figure S1.tif]
